# The Role of HECT-Type E3 Ligase in the Development of Cardiac Disease

**DOI:** 10.3390/ijms22116065

**Published:** 2021-06-04

**Authors:** Jun Goto, Yoichiro Otaki, Tetsu Watanabe, Masafumi Watanabe

**Affiliations:** Department of Cardiology, Pulmonology and Nephrology, Yamagata University School of Medicine, 2-2-2 Iida-Nishi, Yamagata 990-9585, Japan; j-goto007008@med.id.yamagata-u.ac.jp (J.G.); tewatana@med.id.yamagata-u.ac.jp (T.W.); m-watanabe@med.id.yamagata-u.ac.jp (M.W.)

**Keywords:** ubiquitylation, HECT-type E3 ligase, cardiac disease

## Abstract

Despite advances in medicine, cardiac disease remains an increasing health problem associated with a high mortality rate. Maladaptive cardiac remodeling, such as cardiac hypertrophy and fibrosis, is a risk factor for heart failure; therefore, it is critical to identify new therapeutic targets. Failing heart is reported to be associated with hyper-ubiquitylation and impairment of the ubiquitin–proteasome system, indicating an importance of ubiquitylation in the development of cardiac disease. Ubiquitylation is a post-translational modification that plays a pivotal role in protein function and degradation. In 1995, homologous to E6AP C-terminus (HECT) type E3 ligases were discovered. E3 ligases are key enzymes in ubiquitylation and are classified into three families: really interesting new genes (RING), HECT, and RING-between-RINGs (RBRs). Moreover, 28 HECT-type E3 ligases have been identified in human beings. It is well conserved in evolution and is characterized by the direct attachment of ubiquitin to substrates. HECT-type E3 ligase is reported to be involved in a wide range of human diseases and health. The role of HECT-type E3 ligases in the development of cardiac diseases has been uncovered in the last decade. There are only a few review articles summarizing recent advancements regarding HECT-type E3 ligase in the field of cardiac disease. This study focused on cardiac remodeling and described the role of HECT-type E3 ligases in the development of cardiac disease. Moreover, this study revealed that the current knowledge could be exploited for the development of new clinical therapies.

## 1. Introduction

Despite advances in medicine, cardiovascular disease remains a significant public health problem associated with high mortality [1,2]. Heart failure (HF) is a major cause of cardiovascular deaths. Maladaptive cardiac remodeling caused by hypertension, ischemic heart disease, and other cardiac diseases is accompanied by complex mechanisms that lead to the development of HF [3,4]. Further studies are needed to prevent maladaptive cardiac remodeling and subsequent heart failure.

The modification of eukaryotic proteins with ubiquitin, named ubiquitylation, controls their lifetimes, abundance, localization, interactions, and activities, thereby regulating protein function at all levels. Thus, ubiquitylation plays a pivotal role in a wide range of cellular processes, such as signal transduction, transcriptional regulation, and maintenance of homeostasis. Failing hearts from patients with dilated cardiomyopathy and those with ischemic heart disease show hyper-ubiquitylation compared to donor hearts [5]. An increase in key components of ubiquitylation (ubiquitin, ubiquitin-activating enzyme (E1), ubiquitin-conjugating enzyme (E2), and some ubiquitin ligase (E3)) and a decrease in deubiquitinating enzymes have been observed [5,6,7]. Furthermore, several cardiac diseases, such as cardiac amyloidosis, hypertrophic cardiomyopathy, and hereditary cardiomyopathy, impair the ubiquitin–proteasome system [8,9]. The overall observed change in the ubiquitylation cascade in failing hearts is considered an adaptive response to an increased protein burden derived from increased protein synthesis that accompanies the hypertrophic response or an excess of damaged or modified proteins to be targeted for proteasomal degradation.

Ubiquitin E3 ligase is pivotal in conferring specificity to ubiquitylation and provides particularly interesting targets for therapeutic interventions. In the 2000s, many studies focused on the cardiac ubiquitin E3 ligases to clarify the role of ubiquitylation in the development of cardiac diseases, such as the carboxyl terminus of Hsp70 interacting protein (CHIP), atrogen-1, muscle ring finger (MuRF) family, mouse double mutant 2 homolog (MDM2), cellular inhibitor of apoptosis, casitas b-lineage lymphoma, and E6-associated protein (E6AP) [10,11,12,13,14,15,16,17]. Cardiac ubiquitin E3 ligase plays several roles in protein turnover, energy metabolism, receptor internalization, hypertrophic response, apoptosis, and tolerance to ischemia/reperfusion (I/R) in cardiomyocytes [18,19]. Although elevated expression levels of E6AP were observed in mice after pressure overload [16], the functional role of E6AP has never been examined. Thus, our knowledge of the molecular mechanism of HECT-type E3 ligase in the development of cardiac disease is still lacking.

HECT-type E3 ligase is reported to be involved in a wide range of human diseases and health including neurodegenerative diseases, neurological syndromes, and cancers [20,21,22]. Notably, it is considered as an intriguing target in drug discovery in the context of cancer biology [23]. HECT-type E3 ligases are highly conserved between cells and tissues; as a result, it is tempting to speculate that they also contribute to human cardiac health and disease. The role of HECT-type E3 ligases in the development of cardiac diseases has been examined and uncovered in the last decade. There are only a few review articles summarizing recent advancements regarding HECT-type E3 ligase in the field of cardiac disease [24]. This study focused on cardiac remodeling and described the role of HECT-type E3 ligases in the development of cardiac disease. Moreover, this study revealed that the current knowledge can be exploited for the development of new clinical therapies.

## 2. Ubiquitylation

### 2.1. Ubiquitylation

Ubiquitylation is a post-translational modification that covalently conjugates the ubiquitin molecule through the C-terminus to a lysine residue on a substrate protein. Ubiquitylation results in the turnover of the ubiquitylated substrate protein by either the proteasome or lysosome, a change in subcellular localization of the substrate protein, or alteration of substrate protein function [25]. Ubiquitylation is mediated by three enzymes and scaffolding proteins: E1, E2, and E3. There are only few E1s and several E2s; however, E3 ligases constitute a large class of proteins with the human genome encoding more than 600 putative E3 ligases and E3 ligase complexes [26,27,28,29]. Therefore, the specificity of ubiquitylation is determined by the numerous E3 ligases that recognize a specific substrate protein [30]. E3 ligases are also modulators of the rate-limiting step in this enzymatic cascade, participating in substrate protein recognition and catalytic transfer of ubiquitin.

**Figure 1:**
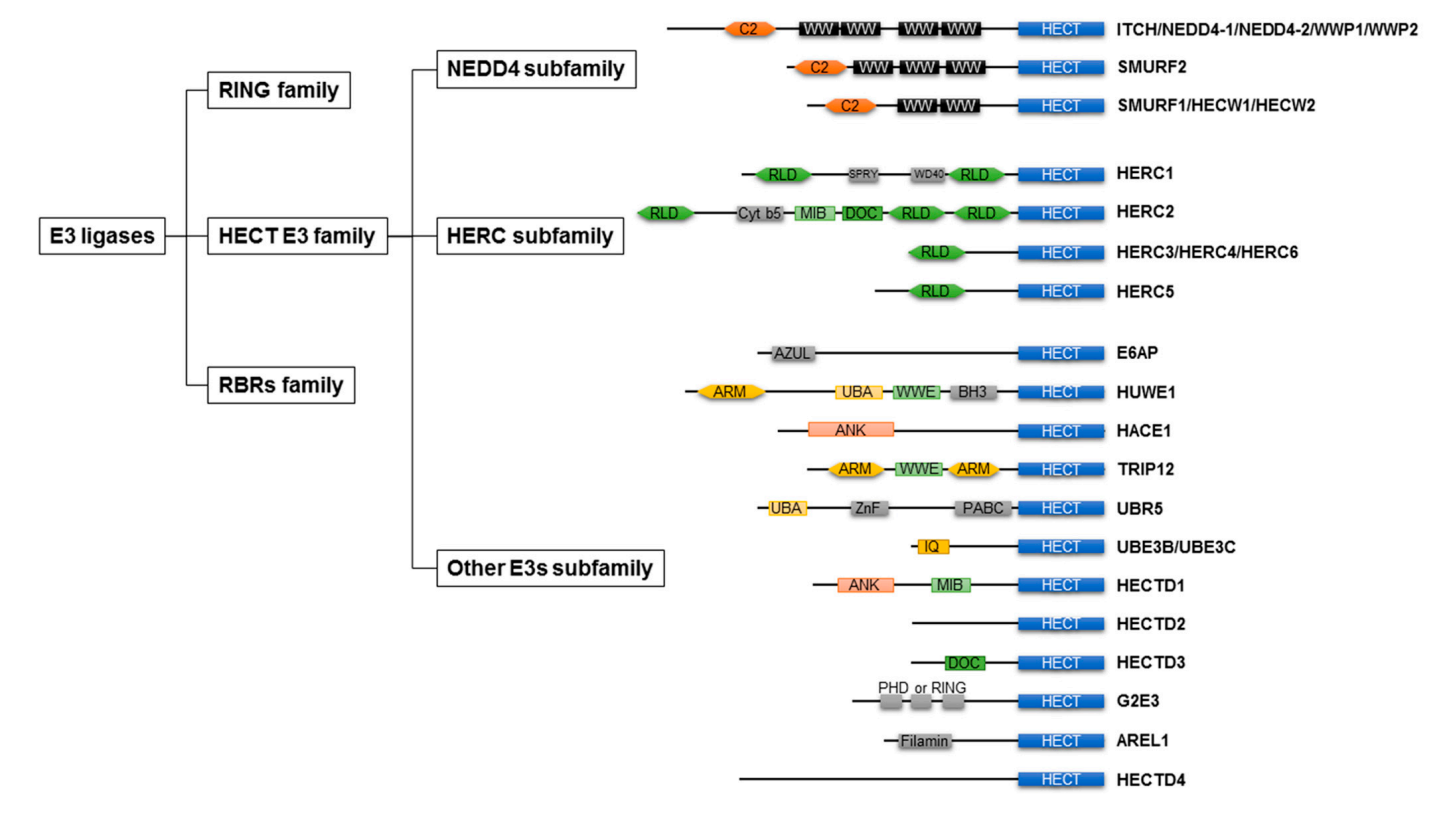
Classification of ubiquitin E3 ligases and the domain architecture of HECT-type E3 ligases. E3 ligases are classified into three groups: RING family, HECT E3 family, RBRs family. HECT E3 family are grouped into three subfamilies: NEDD4 subfamily, HERC subfamily, other E3 subfamily. RING, really interesting new genes; HECT, homologous to E6AP C-terminus; RBRs, RING-between-RINGs; NEDD4, neural precursor cell expressed developmentally downregulated 4; HERC, HECT and RLD domain containing E3 ubiquitin protein ligase; WWP, WW domain containing E3 ubiquitin protein ligase; SMURF, SMAD ubiquitin regulatory factor; HECW, HECT, C2, and WW domain containing E3 protein ligase; E6AP, E6-associated protein; HUWE1, HECT, UBA, and WWE domain containing E3 ubiquitin protein ligase 1; HACE1, HECT domain and ankyrin repeat containing E3 ubiquitin protein ligase 1; TRIP12, thyroid hormone receptor interactor 12; UBR5, ubiquitin protein ligase E3 component N-recognin 5; UBE3B, ubiquitin–protein ligase E3B; UBE3C, ubiquitin protein ligase E3C; HECTD, HECT domain E3 ubiquitin protein ligase; G2E3, G2/M phase-specific E3 ubiquitin protein ligase; AREL1, apoptosis-resistance E3 ubiquitin protein ligase 1; C2, C2 domain; WW, WW domain; RLD, RCC-like domain; ARM, armadillo repeat; UBA, UBA domain; WWE, WWE domain; ANK, ankyrin repeat.

As shown in Figure 1, E3 ligases are classified into three groups: really interesting new genes (RING), homologous to E6AP C-terminus (HECT), and RING-between-RINGs (RBRs). The domain architecture and mechanism of ubiquitylation depend on the class of E3 ligases [31].

Substrate proteins are modified by a single ubiquitin moiety on one or multiple sites, giving rise to mono- and multi-mono-ubiquitylated proteins, respectively. In addition, a wide variety of polyubiquitin chains can be formed on substrate proteins, in which the ubiquitin moieties can be linked through either one of the seven internal lysine residues (Lys6, Lys11, Lys27, Lys29, Lys33, Lys48, and Lys63) in ubiquitin or through its N-terminal amino group.

Polyubiquitylation through the Lsy48-linked ubiquitin chain is generally used for the ubiquitin–proteasomal degradation pathway. Substrate proteins that receive Lys48-linked polyubiquitin chains migrate to and are degraded by the 26S proteasome. The ubiquitin–proteasome system is a protein quality and quantity control system that mediates approximately 80–90% of intracellular protein degradation under optimal nutritional conditions [32,33,34,35,36]. Furthermore, mono-ubiquitylation of lysine residues or polyubiquitylation through Lys63-linked ubiquitin chains are used for nonproteolytic pathways such as DNA repair, relocalization, modifying activity (signal transcriptional activity), or endocytosis [37,38,39]. The consequences for the modified substrate are determined by the type of ubiquitin modification it receives [40].

RING E3 ligases catalyze the direct transfer of ubiquitin from the E2 conjugating enzyme to the substrate, suggesting that the linkage type of the ubiquitin chain is determined by the E2 conjugating enzyme. In contrast to RING-type E3 ligases, HECT-type E3 ligases include an active-site cysteine in the HECT domain, which forms an intermediate thioester bond with ubiquitin before it is conjugated to the substrate protein [41,42]. HECT-type E3 ligase has enzymatic activity and directly catalyzes the covalent attachment of ubiquitin to substrate proteins; therefore, it could determine the linkage type of ubiquitin chain preferred [43].

### 2.2. HECT-Type E3 Ligase

In 1995, HECT-type E3 ligases were found in all eukaryotic organisms and ubiquitin [41]. E6AP transcribed from the ubiquitin–protein ligase E3A gene was the first identified HECT-type E3 ligase, leading to the discovery of the HECT-type E3 ligase family [41,44,45]. There are 28 types of HECT-type E3 ligases in humans [46], which are commonly grouped into three groups based on the presence of distinct amino acid sequence motifs or domains within the N-terminal: NEDD4 subfamily, HERC subfamily, and other HECT-type E3 ligases [47] (Figure 1). The HECT domain is an approximately 40 kDa domain positioned at the C-terminus of the E3 ligases, consisting of the N-lobe and C-lobe. The N-lobe represents the E2 binding domain, whereas the C-lobe contains an active site cysteine to receive ubiquitin. In the HECT family, 16–92% amino acid identity was found for this domain [48]. The domain architecture of the HECT-type E3 ligases is shown in Figure 1.

#### 2.2.1. NEDD4 Subfamily

The NEDD4 subfamily member includes nine types of HECT-type E3 ligases and accounts for approximately 30% of HECT-type E3 ligases [22]. The NEDD4 family is characterized by the presence of C2 and 2–4 WW domains. The N-terminal C2 domain is defined as a Ca^+^ phospholipid binder [49]. The WW domains are responsible for recognizing substrates and have also been found to form intramolecular interactions with the HECT domain of the E3 ligases [50,51]. Some NEDD4 subfamily members are often expressed as alternative splice isoforms [47].

#### 2.2.2. HERC Subfamily

The HERC subfamily is characterized by a HECT domain and one or more regulators of chromosome condensation-like domains (RLDs), an effector protein domain that was first identified as a regulator of chromosome condensation 1 [52]. In humans, the HERC subfamily comprises six members, which can be further organized into two large and four small HERCs. Large HERCs (HERC1 and 2) have two or three RLDs; however, small HERCs (HERCs 3, 4, 5, and 6) have one RLD. RLD has dual functions: one side of the domain acts as a guanine nucleotide exchange factor for the small GTPase Ran, whereas the opposite side interacts with chromatin through histones H2A and H2B [53,54].

#### 2.2.3. Other HECT E3s

Each member of another HECT E3 ligase lacks WW or RLD domains and has a distinct variety of N-terminal domains. There are several N-terminal domains of other HECT E3 ligases, such as WWE and armadillo repeats (HUWE1 and TRIP12), UBA domain (HUWE1 and UBR5), ankyrin repeats (HACE1 and HECTD1), and IQ motifs (UBE3B and UBE3C) [55]. In addition, the MIB domain in HECTD1 and the DOC domain in HECTD3 were common in the N-terminus of HERC2.

## 3. Importance of Ubiquitylation in Cardiac Disease

The adult heart endures a wide range of physiological and pathophysiological stresses during life. Accumulating evidence indicates that ubiquitylation is involved in developing cardiac diseases [17,56,57,58,59,60]. Cardiac proteins are in a dynamic state of continual degradation and resynthesis and are thought to replace all in 30 days under normal circumstances. Protein turnover is critical to cardiomyocytes as post-mitotic cells with minimal regenerative capacity because protein aggravation is cytotoxic [61,62]. Eukaryotic cells have developed multilayered protein quality control mechanisms primarily carried out by chaperones, lysosomal autophagy, and the ubiquitin–proteasome system [63]. More than 70% of the protein turnover is regulated by the ubiquitin–proteasome system [64,65,66]. An experimental study demonstrated that the balance of protein turnover could lead to protein accumulation and aggravation during cardiac remodeling [67]. The discovery that cardiac ubiquitin E3 ligases, such as muscle-specific ubiquitin ligase atrogin-1 and MuRF family, yields cardiac growth, and remodeling through sarcomeric protein turnover, indicated that the ubiquitylation cascade is fundamental to the maintenance of normal cardiac function through protein quality control [17,68,69].

As previously mentioned, ubiquitylation is involved in most aspects of eukaryotic cell biology, such as intracellular signaling, transcriptional control, and regulation of cell death. Research regarding the role of cardiac ubiquitin E3 ligases has developed from protein turnover to cellular processes such as signal transduction, transcriptional regulation, maintenance of homeostasis, mitochondrial dynamics, receptor turnover, and energy metabolism [17,19]. In the following paragraph, we will limit ourselves to a discussion of HECT-type E3 ligases that have been associated with cardiac diseases.

## 4. Cardiac Hypertrophy and HECT-Type E3 Ligase

Pathological cardiac hypertrophy caused by hypertension, aortic stenosis, and other disease-related stresses is an early milestone during the clinical course of HF [70]. A study detected an accumulation of ubiquitylated proteins and abnormal aggregation in cardiomyocytes collected from patients with decompensated cardiac hypertrophy [71]. Pressure overload-induced cardiac hypertrophy is associated with a marked increase in protein synthesis at a rate exceeding degradation with an increase in ubiquitin, E2, and several E3 ligases [16,72,73,74], indicating hyper-ubiquitylation in rat and mouse models of hypertrophy.

At the cellular level, cardiomyocyte hypertrophy is characterized by an increase in cell size, enhanced protein synthesis, multiplication of sarcomeres, a switch of proteins and enzymes to fetal isoforms, changes in intracellular Ca^2+^ handling, metabolic alterations, and increased rates of apoptosis [75,76]. Numerous key regulatory pathways that promote cardiac hypertrophy are either targets or components of the ubiquitylation cascade. Several signaling pathways contribute to cardiac hypertrophy, such as Wnt/β catenin, calcineurin/nuclear factor of activated T-cells (NFAT), the Janus kinase (JAK)/signal transduction and the activator of transcription (STAT) pathway [77,78,79].

This study shows the current understanding of HECT-type E3 ligases in the development of cardiac hypertrophy. An overview of HECT-type E3 ligases in cardiac hypertrophy is summarized in Table 1.

### 4.1. ITCH

The ubiquitin E3 ligase ITCH is a 903 amino acid residue protein that contains an N-terminal C2 domain, four WW domains, and an HECT domain [55]. ITCH was originally identified after genetic analysis of a mutant mouse with aberrant immunological phenotypes and constant skin scratching [94]. The WW domain recognizes the proline-rich PPXY consensus sequence in substrate proteins, and the HECT domain attaches ubiquitin molecules to substrates [95]. Thioredoxin-interacting protein, disheveled (Dvl), c-jun, Jun-B, Notch, Gli, p73, p63, cellular FLICE-inhibitory protein, B cell lymphoma/leukemia 10, Forkhead box O1, transforming growth factor-β-activated kinase 1, and ITCH are known substrates of ITCH for degradative ubiquitylation. Frizzled receptor 4, phospholipase C gamma 1, protein kinase C-θ, Deltex, ErbB4, CXC chemokine receptor 4, and transient receptor potential vanilloid 4 are known substrates of ITCH for degradative ubiquitylation by the non-ubiquitin–proteasome system. Furthermore, small mothers against decapentaplegic (SMAD2) are known substrates of ITCH for non-degradative ubiquitylation [96,97].

**Figure 2:**
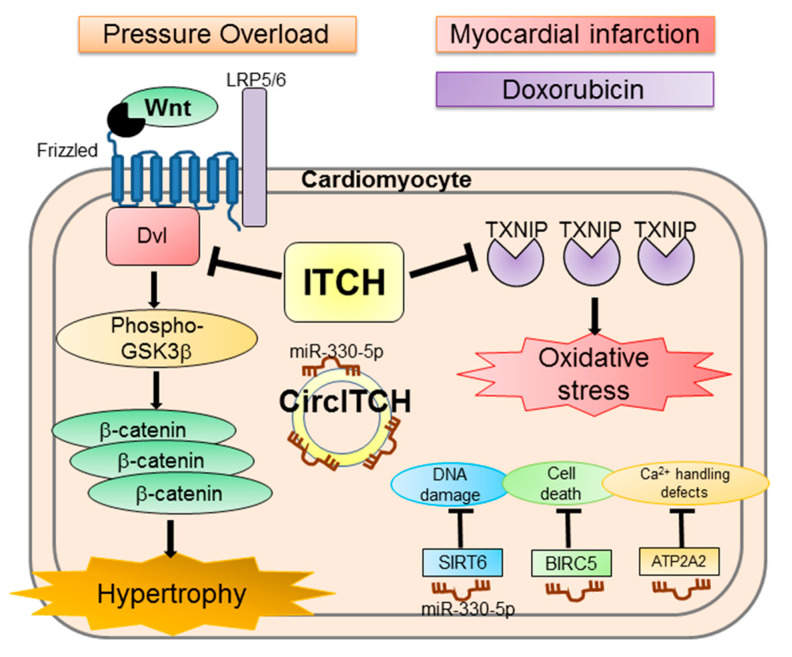
A simplified model depicting the function of ITCH in the development of cardiac hypertrophy, myocardial infarction, and doxorubicin-induced cardiomyopathy. ITCH targets disheveled protein for ubiquitin-proteasome degradation, resulting in inhibition of the Wnt/β-catenin signaling pathway; ITCH attenuates cardiac hypertrophy induced by pressure overload. ITCH targets thioredoxin-interacting protein for ubiquitin–proteasome degradation and ameliorates reactive oxygen species-induced cardiotoxicity through the thioredoxin system in myocardial infarction and doxorubicin-induced cardiomyopathy models. Circ ITCH absorbs miR-330-5p, resulting in the activation of miR-330-5p targets (SIRT6, BIRC5, and ATP2A2), thereby ameliorating doxorubicin-induced cardiomyopathy by reducing DNA damage, cell death, and calcium handling defects. LRP5/6, low-density lipoprotein receptor-related protein 5/6; Circ ITCH, circular RNA ITCH; miR, microRNA; SIRT6, sirtuin 6; BIRC5, baculoviral IAP repeat containing 5; ATP2A2, ATPase sarcoplasmic/endoplasmic reticulum Ca^2+^ transporting 2; Ca^2+^, calcium.

Accumulating evidence demonstrates that Wnt/β-catenin signaling is activated after transverse aortic constriction (TAC) and induces pathological cardiac hypertrophy [79,98,99,100]. Dvl is an intracellular principle component of signaling [101]. Dvl has approximately 700 amino acids, harboring conserved DIX, basic and serine threonine-rich region, PDZ, proline-rich PPXY consensus sequence region, and DEP domains. We previously reported that key molecules of the Wnt/β-catenin signaling pathway after TAC are inhibited in cardiac-specific overexpression of ITCH transgenic mice. In this study, ITCH targets Dvl proteins for ubiquitin–proteasome degradation in cardiomyocytes and attenuates cardiac hypertrophy by suppressing the Wnt/β-catenin signaling pathway (Figure 2) [80].

ITCH potentially mediates the degradation of non-Dvl substrates during cardiac hypertrophy [102,103]; therefore, further studies are needed to clarify the effect of ITCH on other substrates during cardiac hypertrophy. Overall, ITCH may be a therapeutic target for cardiac hypertrophy. 

### 4.2. NEDD4-2

NEDD4-2, the most ancient member of the NEDD4 subfamily, was originally identified in a screening of genes downregulated during development of the central nervous system [104]. NEDD4-2 consists of a C2 domain, four WW domains, and an HECT domain. NEDD4-2 is a multifunctional protein whose mutations are associated with development disorders, hypertension, epilepsy, and end-stage renal disease [105,106]. NEDD4-2 has been suggested to function as an E3 ligase for several PY motif-containing proteins such as SMADs, Dvl2, epithelial Na^+^ channel (ENaC), voltage-dependent cardiac Na^+^ channel (Nav1.5), KCNQ1 potassium channel [107], and human ether-a-go-go-related gene [108,109].

It was reported that high salt diet-induced cardiac hypertrophy and systolic function were exacerbated in NEDD4-2 deficient mice with higher expression levels of ENaC in the kidney [82]. Furthermore, Galiana-Simal et al. demonstrated that NEDD4-2 is phosphorylated; therefore, NEDD4-2 functions to ubiquitylate ENaC in hypertrophied myocardium of aldosterone-treated rats [81]. These findings suggest an association between NEDD4-2 and cardiac hypertrophy.

### 4.3. WWP2

The WW domain-containing E3 ubiquitin protein ligase 2 (WWP2), also known as atrophin-1-interacting protein 2, was originally identified in screening for WW domain-containing proteins [110]. WWP2 is ubiquitously expressed in the heart, placenta, lung, liver, muscle, kidney, pancreas, and brain [111]. There are three isoforms in WWP2: a full-length WWP2, an N-terminal isoform presumably generated by failure to splice-out intron 9-10, and a C-terminal isoform possibly generated from a second promoter within introns 10-11. WWP2 interacts with multiple substrates, such as phosphatase and tensin homolog deleted from chromosome 10 (PTEN), SMADs, Oct4, and ENaC [112,113]. WWP2 is a multifunctional E3 ubiquitin ligase, which is involved in palatogenesis, craniofacial development, innate immune response, tumorigenesis, and cell death [114,115,116,117,118,119]. WWP2 was also reported to regulate PTEN/ phosphatidylinositol 3-kinase (PI3K)/Akt signaling and transforming growth factor-beta (TGF-β)/SMAD signaling [119].

**Figure 3:**
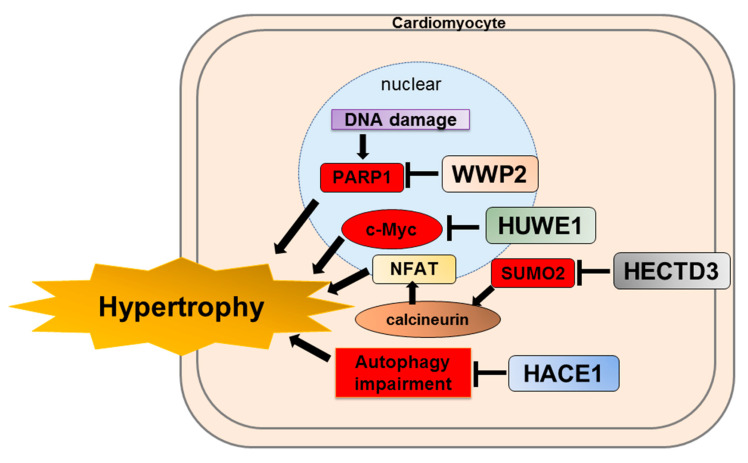
Schematics depicting the protective role of HECT-type E3 ligases for cardiac hypertrophy. WWP2 targets PARP1 for ubiquitin-proteasome degradation. HUWE1 targets c-Myc for ubiquitin–proteasome degradation. HECTD3 targets SUMO2 for ubiquitin–proteasome degradation. HACE1 ameliorates autophagy impairment. These HECT-type E3 ligases are a protective role for cardiac hypertrophy. WWP2, WW domain-containing E3 ubiquitin protein ligase 2; PARP1, Poly (ADP-ribose) polymerase-1; HUWE1, HECT, UBA, and WWE domain containing E3 ubiquitin protein ligase 1; HECTD3, HECT domain E3 ubiquitin protein ligase 3; SUMO2, small ubiquitin-like modifier 2; NFAT, nuclear factor of activated T-cells; HACE1, HECT domain and ankyrin repeat containing E3 ubiquitin protein ligase 1.

Isoproterenol is a chronic infusion of β-stimulant, which induces cardiac hypertrophy and fibrosis, leading to heart failure. Poly(ADP-ribose) polymerase-1 (PARP1) is a critical injury factor in cardiac remodeling. Increases in PARP1 and poly(ADP-ribosyl)ation (PARylatin; PARP1 activity) have been observed in cardiac remodeling, leading to extreme energy consumption by myocardial cells [120,121]. PARP1 is activated to generate PARylation via recognition of damaged DNA fragments and induces cardiomyocyte damage, leading to apoptosis and necrosis [122]. Zhang N et al. reported that full-length WWP2 knockout mice exacerbated cardiac systolic function, hypertrophy, and fibrosis compared to WT mice after isoproterenol infusion. In addition, the protein expression of WWP2 decreased; however, PARP1 increased in response to isoproterenol in vitro and in vivo. WWP2 interacts with PARP1 and degrades it through the ubiquitin–proteasome system by regulating ubiquitylation of PARP1 K418 and K249 sites in the BRCT domain, leading to the inhibition of isoproterenol-induced cardiac remodeling (Figure 3) [84]. WWP2 is a protective factor against isoproterenol-induced cardiac remodeling.

### 4.4. HUWE1

The ubiquitin E3 ligase HECT, UBA, and WWE domain containing E3 ubiquitin protein ligase 1 (HUWE1), also termed UREB1, HECTH9, ARF-BP1, MULE, E3 Histone, and LASU1, is a 4347 amino acid residue protein that contains an N-terminal armadillo repeat domain, a UBA domain, a WWE domain, a BH3 domain, and a HECT domain [55]. c-Myc, p53, and myeloid cell leukemia-1 are known substrates of HUWE1 for degradative ubiquitylation [123]. In contrast, Dvl and herpesvirus-associated ubiquitin-specific proteases are known substrates of HUWE1 for non-degradative ubiquitylation [124,125]. HUWE1 is associated with proliferation, differentiation, apoptosis, DNA repair, and stress response [124]. HUWE1 has a general preference for attaching the K48 polyubiquitin chain to substrate proteins [126,127].

Recently, Dadson et al. reported that HUWE1 expression was reduced in the left ventricle of patients with end-stage heart failure. Furthermore, conditional cardiac-specific HUWE1 knockout mice develop spontaneous cardiac hypertrophy, left ventricular dysfunction, and premature death with an increase in c-Myc in the heart (Figure 3) [85]. In addition, conditional cardiac-specific HUWE1 knockout mice showed impaired mitochondrial energy metabolism and reactive oxygen species defense, accompanied by reduced protein expression levels of key regulators such as Pgc-1α and Pink1 [128,129,130]. Transcriptomic analysis of HUWE1 knockout mice revealed that elevated c-Myc directly inhibits the transcription of Pgc-1α and Pink1. Cardiac hypertrophy and left ventricular dysfunction were diminished in conditional cardiac-specific HUWE1 and c-Myc double knockout mice. Therefore, HUWE1 in the heart could be a potential therapeutic target for cardiac hypertrophy through its interaction with c-Myc.

### 4.5. HACE1

The ubiquitin E3 ligase HECT domain and ankyrin repeat containing E3 ubiquitin protein ligase 1 (HACE1) is a 909 amino acid residue protein that contains an N-terminal ankyrin repeat domain and an HECT domain [55]. HACE1 was discovered as a chromosome 6q21 tumor-suppressor gene in Wilms’ tumors [131]. Subsequently, HACE1 is noted as a tumor suppressor in multiple cancers [132]. As a mechanism of tumor suppression, it has been shown to catalyze the ubiquitylation of Ras-related C3 botulinum toxin substrate 1, a potent oncogene [133].

Zhang et al. demonstrated that HACE1 gene expression is upregulated in dilated cardiomyopathy compared with non-failing hearts in silico. They demonstrated that HACE1 deficiency in mice exacerbated heart failure and increased mortality after severe TAC. HACE1 knockout mice show abnormal cardiac hypertrophy, left ventricular dysfunction, accumulation of LC3, p62, and ubiquitylated proteins enriched for cytoskeletal species (Figure 3) [86]. This study reveals that HACE1 is required to fuse autophagosomes with lysosomes, independent of its E3 ligase activity. Hence, HACE1 may play an important role in suppressing protein accumulation and aggravation by regulating autophagy during cardiac remodeling.

### 4.6. HECTD3

The ubiquitin E3 ligase HECT domain E3 ubiquitin protein ligase 3 (HECTD3) is an 861 amino acid residue protein that contains a DOC domain and an HECT domain. Trio-associated repeats on actin and RAF proto-oncogene serine/threonine-protein kinase are known substrates of HECTD3 for degradative ubiquitylation by the ubiquitin–proteasome system. Furthermore, mucosa-associated lymphoid tissue proteins 1, caspase-8, caspase-9, and STAT3 are substrates of HECTD3 for non-degradative ubiquitylation [134,135].

Small ubiquitin-like modifier 2 (SUMO2) is the most efficient activator of calcineurin/NFAT signaling to induce cardiac hypertrophy [136]. Furthermore, the inflammatory response is associated with myocardial fibrosis and hypertrophy [78,137]. In cardiac and other systems, the JAK/STAT signaling pathway is associated with the inflammatory response [138]. Recently, Rangrez et al. demonstrated that HECTD3 targets SUMO2 for ubiquitin–proteasome degradation and suppresses calcineurin/NFAT signaling (Figure 3). In addition, they showed that HECTD3 reduced the activation of STAT1 by attenuating its phosphorylation through the induction of its polyubiquitylation. In this study, AAV9-mediated overexpression of HECTD3 in mice reduced cardiac SUMO2/STAT1 levels, pathological hypertrophy, largely abolished macrophage infiltration, and fibrosis induced by pressure overload [87]. Thus, HECTD3 may be a potential therapeutic target for cardiac hypertrophy.

## 5. Cardiac Fibrosis and HECT-Type E3 Ligase

Pathological cardiac fibrosis is a process characterized by excessive deposition of extracellular matrix (ECM), leading to the development of cardiac dysfunction, arrhythmia, and HF [139,140,141]. Several pathophysiological conditions induce cardiac fibrosis, such as pressure overload, volume overload, myocardial infarction, dilated and hypertrophied cardiomyopathy, various toxic insults, metabolic disturbances, and aging [142,143,144,145].

Cardiac fibroblasts are key effector cells in cardiac fibrosis and are responsible for ECM homeostasis in the heart [139]. After cardiac fibroblasts are activated by regulators of tissue fibrosis, such as angiotensin II, connective tissue growth factor, bone morphogenetic protein (BMP), Wnt ligands, cytokines, and TGF-β superfamily, they differentiate into myofibroblasts with an increase in ECM protein [146]. TGF-β1 contributes to cardiac fibrosis development through SMAD-dependent and SMAD-independent pathways. TGF-β1 generally exerts its biological effects by activating downstream mediators, including SMAD2 and SMAD3, while negatively regulated by SMAD7 expression [147,148,149]. SMADs have been reported to play a pivotal role in the transcription of ECM proteins [150].

Although the pathophysiological conditions leading to cardiac fibrosis are different from those of cardiac diseases, it is valuable to explore the common mechanisms involved in cardiac fibrosis. This study shows the current understanding of the role of HECT-type E3 ligases in cardiac fibrosis. An overview of HECT-type E3 ligases in cardiac fibrosis is summarized in Table 1.

### 5.1. WWP2

An outline of the domain architecture, function, and substrates of WWP2 is described in the previous paragraph. Chen et al. identified the WWP2 N-terminal isoform as a positive regulator of the pro-fibrotic gene network associated with cardiac fibrosis using systems genetics in human and murine dilated cardiomyopathy and repaired tetralogy of Fallot. The WWP2 N-terminal isoform consists of a C2 domain and a WW domain, indicating the absence of the HECT domain. The left ventricular single-cell RNA sequence indicated that WWP2 is mainly expressed in fibroblasts, immune cells, and endothelial cells. WWP2^mut/mut^ mice lacking the N-terminal isoform and full-length WWP2 attenuated cardiac fibrosis and preserved cardiac function after angiotensin II infusion or myocardial infarction. The N-terminal region of WWP2 interacts with SMADs potentially through its mono-ubiquitylation and mediates the TGF-β1-induced nucleocytoplasmic shuttling and transcriptional activity of SMAD2 [88,151]. Thus, WWP2 is an important regulator of pro-fibrotic and ECM genes. These findings provide new understanding into the role of HECT-type E3 ligases independently of the HECT domain.

### 5.2. SMURF1

SMAD ubiquitin regulatory factor (SMURF) was initially identified as a regulator of SMAD1 stability [152]. SMURFs are composed of two members, SMURF1 and SMURF2. SMURFs have been implicated in determining the competence of cells in response to the TGF-β/BMP signaling pathway [153]. SMURF1 consists of a C2 domain, two WW domains, and an HECT domain. SMURF is a multifunctional protein that is involved in cell cycle progression, cell proliferation, differentiation, DNA damage response, and maintenance of genomic stability. SMURF1 targets Dvl2, SMADs, RhoA, and Runx2, 3 [154]. SMURF1 modulates several signal transduction pathways, such as the TGF-β/BMP signaling pathway, Wnt signaling, mitogen-activated protein kinase signaling, and RhoA/Rho-associated kinase signaling [155]. SMURF1 plays an important role in heart development, including outflow tract septation and cell-type specification, by controlling cilium-associated BMP signaling [156,157].

BMP-2, as a novel fibrosis-antagonizing cytokine, have a potential beneficial effect in attenuating pressure overload-induced cardiac fibrosis. Wang S et al. demonstrated that SMURF1 interacted with SMAD6 and that this SMURF1/SMAD6 complex was involved in BMP2 antagonization of TGF-β1 mediated protein kinase C-δ and SMAD3 signaling in cardiomyocytes [89]. This finding suggests that SMURF1 may contribute to cardiac fibrosis development.

Endothelial colony-forming cells have been reported to reduce cardiac fibrosis in myocardial infarction due to their proliferation and secretion of exosomes, which transfer microRNAs. Cardiac fibroblast activation is ameliorated by exosomes from endothelial colony-forming cells treated with normoxia compared to those treated with hypoxia. Liu et al. found that miR-10b-5p was enriched in exosomes from normoxia and targeted SMURF1 and histone deacetylase 4 using next-generation RNA sequencing. Thus, inhibition of mRNA expression of SMURF1 by miR-10b-5p was suggested to participate in the antifibrotic effects of exosomes derived from endothelial colony-forming cells treated with normoxia [90].

### 5.3. SMURF2

SMURF2 consists of a C2 domain, three WW domains, and an HECT domain. SMURF2 targets SMADs, heat shock proteins 27, and p53 [154]. SMURF2 was reported to be downregulated in DCM [158]. SMURF functions as a mediator of TGF-β signaling via interaction with SMAD7 containing PY motif during cardiac fibrosis [91].

Meng et al. examined the effect of a SMAD3 inhibitor on angiotensin II-induced cardiac fibrosis. The protein expression level of SMURF2 in the mouse heart increased, while that of SMAD7 decreased after angiotensin II administration. However, this effect was reversed by the SMAD3 inhibitor, suggesting that the SMAD3 inhibitor protected cardiac SMAD7 from SMURF2-mediated ubiquitin–proteasome degradation. Since SMAD7 functions as an inhibitor of both TGF-β/SMAD and NF-κB signaling, an increase in cardiac SMAD7 could be another mechanism through which SMAD3 inhibitor blocked SMAD3-mediated cardiac fibrosis and NF-κB-driven cardiac inflammation [92]. This finding suggests that SMRUF2 may contribute to the development of cardiac fibrosis.

## 6. HECT-Type E3 Ligase and HF with Preserved Ejection Fraction

HF is classified into three groups: HF with reduced ejection fraction, HF with mid-range ejection fraction, and HF with preserved ejection fraction (HFpEF) [159]. The prevalence rate of HFpEF has been estimated to range from one-third to one-half of all HF patients and is projected to increase [160,161]. Aging is associated with progressive fibrosis, leading to the development of HFpEF [162]. HFpEF in older persons is typified by a broad range of cardiac and non-cardiac abnormalities and reduced reserve capacity in multiple organ systems [163]. To date, there are no approved therapies available for reducing mortality or hospitalization for these patients due to the heterogeneity of HFpEF [164]. This study demonstrates the role of HECT-type E3 ligases in HFpEF development.

### WWP1

The WW domain containing E3 ubiquitin protein ligase 1 (WWP1) is a multifunctional protein containing an N-terminal C2 domain, four tandem WW domains for substrate binding, and a C-terminal catalytic HECT domain for ubiquitin transfer. WWP1 was reported to be associated with cancer, aging, neurological disorders, and bone homeostasis [165,166]. WWP1 has been suggested to function as an E3 ligase for several PY motif-containing proteins such as connexin 43, large tumor suppressor 1 and 2, SMAD2, Krüppel-like transcription factor 5, p63, ErbB4/HER4, Runx2, JunB, atrophin-1, and several non-PY motif-containing proteins such as TGF-β receptor 1, SMAD4, Krüppel-like transcription factor 2, and p53 [167]. WWP1 modulates several signal transduction pathways, such as TGF-β signaling, epidermal growth factor signaling, and apoptosis signaling [166].

WWP1 is reported to be highly expressed in the heart [111,168,169] and increases with aging [166]. It was reported that the WWP1 overexpressed mice showed left ventricular hypertrophy, extracellular matrix remodeling, diastolic dysfunction, except systolic dysfunction, indicating the HFpEF phenotype [93]. The precise mechanism by which HFpEF develops has not yet been fully elucidated. Gene analysis data from RNA sequencing using right ventricular endomyocardial biopsies indicated enrichment in mitochondrial adenosine triphosphate synthesis/electron transport and a decrease in endoplasmic reticulum stress, autophagy, and angiogenesis [170]. The NEDD4 family is involved in the development of HFpEF, suggesting the importance of post-translational modification by ubiquitylation in HFpEF as well as HF with reduced ejection fraction. This knowledge provides new insights into HFpEF physiology. WWP1 may have potential therapeutic relevance in the context of HFpEF.

## 7. Ischemia/Reperfusion Injury and HECT-Type E3 Ligase

Early reperfusion of the ischemic myocardium plays a vital role in minimizing myocardial infarction. However, the effects of reperfusion are complex and include harmful effects, collectively referred to as reperfusion injury [171,172]. The underlying mechanisms of I/R injury are associated with reactive oxygen species generation, intracellular Ca^2+^ disturbance, rapid pH restoration, and inflammation [173]. Therefore, I/R injury is accompanied by detrimental manifestations, including myocardial necrosis and apoptosis [174]. Cell death contributes to an increase in infarct size, and regulation of this mechanism contributes to improved cardiac function [175]. The ubiquitin–proteasome system plays a pivotal role in I/R injury protection in the heart, organ transplantation, and cerebral ischemia [176,177,178]. CHIP has been reported to be required for cardioprotection after myocardial infarction in mice [179]. Furthermore, MDM2, which targets p53 for degradative ubiquitylation, demonstrated a protective role against hypoxia/reoxygenation-induced cell death [15]. Thus, the role of cardiac E3 ligases in the injured myocardium is important. Recently, some HECT-type E3 ligases have been reported to be involved in I/R injury. This study shows the current understanding of the role of HECT-type E3 ligases in I/R injury. An overview of HECT-type E3 ligases in I/R injury is summarized in Table 2.

### 7.1. NEDD4-1

Neural precursor cell expressed developmentally downregulated protein 4-1 (NEDD4-1), also termed NEDD4 and RPF1, is a 1319 amino acid residue protein that contains an N-terminal C2 domain, four WW domains, and an HECT domain [116]. In 1992, NEDD4-1 was discovered in mouse neural precursor cells, whose mRNA levels were downregulated during mouse brain development [104]. To date, the role of NEDD4-1 has been identified as an oncogene, tumor suppressor gene, autophagy regulation, and anti-Parkinson’s disease effect [188,189,190,191]. PTEN, RNA polymerase II, and N-Myc are known substrates of NEDD4-1 for degradative ubiquitylation. In contrast, Akt, MDM2, and α-Synuclein are known substrates of NEDD4-1 for non-degradative ubiquitylation [192]. NEDD4-1 is required for heart development [193]. Activation of the PI3K/Akt pathway regulating cellular processes involved in the cell cycle has been shown to protect the heart from I/R injury [194,195]. PTEN is a tumor suppressor that inhibits PI3K/Akt signaling [196]. NEDD4-1 negatively regulates PTEN stability by catalyzing PTEN polyubiquitylation [188]. Furthermore, NEDD4-1 has been shown to positively regulate the nuclear trafficking of phospho-Akt by K63-linked polyubiquitylation [197]. NEDD4-1 was reported to be downregulated in rat hearts after I/R injury [198]. Recently, it was reported that overexpression of NEDD4-1 ameliorated myocardial apoptosis after I/R injury in rats injected with NEDD4-1 lentivirus vector via activation of PI3K/Akt signaling, suggesting the cardioprotective role of NEDD4-1 [180]. However, NEDD4-1 has numerous other substrates that need to be taken into account when interpreting experimental results because it does not directly demonstrate substrates of NEDD4-1 in the heart.

### 7.2. SMURF2

An outline of the domain architecture, function, and substrates of SMURF2 is described in the previous paragraph. Enhancer of zeste homolog 2 (EZH2) is reported to be one of the SMURF2 substrates. EZH2 is a polycomb group protein associated with pivotal functions, including cell division, embryonic development, and cancer development [199]. EZH2 binds to PTEN, negatively regulates its expression, and upregulates PI3K/Akt signaling [200]. Recently, Dong et al. demonstrated that miR-322/503 plays a beneficial role in myocardial I/R injury. Inhibition of SMURF2 translation induces EZH2 expression and activates PI3K/Akt signaling via miR-322/503, thereby protecting cells from I/R injury [181]. Therefore, SMURF2 might be a potential target for improving the prognosis of myocardial I/R injury.

## 8. Other Cardiac Disease and HECT-Type E3 Ligase

An overview of HECT-type E3 ligases in other cardiac diseases such as doxorubicin cardiotoxicity and arrhythmia is briefly summarized in Table 2.

### 8.1. ITCH and Doxorubicin Cardiotoxicity

The outline of the domain architecture, function, and substrates of ITCH is described in the previous paragraph. Doxorubicin is one of the most widely used anticancer therapies for malignant lymphoma (CHOP therapy), breast cancer (AC therapy), and uterine cancer (AP therapy) [201,202,203]. Although doxorubicin has a significant effect on reducing mortality of these cancer patients, its cumulative and dose-dependent toxicity harms the heart [204]. The mechanisms of doxorubicin cardiotoxicity are involved in inflammation, oxidative stress, apoptosis, mitochondrial impairment, and dysregulation of autophagy [205]. We previously reported that ITCH targets the thioredoxin-interacting protein for ubiquitin–proteasome degradation in cardiomyocytes and ameliorates cardiotoxicity induced by reactive oxygen species, including doxorubicin cardiotoxicity and myocardial infarction (Figure 2). After doxorubicin injection or myocardial infarction, the survival rates were significantly higher in cardiac-specific overexpression of ITCH transgenic mice than in WT littermates [182]. In addition, Zhang et al. demonstrated that miR-34b/c targeted ITCH and suppressed its expression, promoting NFκB and subsequent inflammatory cytokine expression in doxorubicin-treated HL-1 cells [183].

Circular RNAs (circRNAs) are important modulators of cardiac development and disease. Recently, it was reported that circular RNA ITCH derived from 7–14 exons in ITCH acts as a sponge of miR-330-5p, thereby upregulating sirtuin 6, baculoviral IAP repeat containing 5, and ATPase sarcoplasmic/endoplasmic reticulum Ca^2+^ transporting 2 to alleviate doxorubicin cardiotoxicity [185] (Figure 2). Therefore, ITCH and circular RNA ITCH have cardioprotective roles against doxorubicin and could be novel therapeutic targets in doxorubicin cardiotoxicity.

### 8.2. NEDD4-2 and Arrhythmia

An outline of the domain architecture, function, and substrates of NEDD4-2 is described in the previous paragraph. The activity of NEDD4-2 is regulated by Ca^2+^ concentration in cells, and an increase in Ca^2+^ concentration disrupts the autoinhibitory conformation of NEDD4-2, leading to its activation [206].

Nav1.5, which is responsible for the action potential upstroke, is essential for maintaining an adequate conduction velocity of the electrical impulse [207]. It has been reported that protein expression of Nav1.5 is reduced in HF without any change in its mRNA level [208]. Luo et al. uncovered the mechanism of downregulating Nav1.5 in HF. The protein expression of Nav1.5 decreased, while that of NEDD4-2 increased after ionomycin-induced intracellular Ca^2+^ increase. This was reversed by the calcium chelator BAPTA-AM, suggesting an inverse relationship between Nav1.5 and NEDD4-2 expression in neonatal rat cardiomyocytes. Nav1.5 was ubiquitylated and downregulated in NEDD4-2 transfected neonatal rat cardiomyocytes. Reduced expression of Nav1.5 and augmented expression of NEDD4-2 were observed in a volume overload rat heart failure model and cardiomyocytes treated with isoproterenol and angiotensin II. Therefore, calcium-mediated increase in NEDD4-2 induced Nav1.5 ubiquitylation with a resultant reduction in Nav1.5 cardiomyocyte membrane density in HF [186]. Mutations in the gene encoding Nav1.5, SCN5A, have been associated with various arrhythmic disorders such as long QT syndrome, Brugada syndrome, and sick sinus syndrome, indicating the association of NEDD4-2 with cardiac arrhythmia [209,210].

### 8.3. WWP1 and Arrhythmia

An outline of the domain architecture, function, and substrates of WWP1 is described in the previous paragraph. Sudden cardiac death is a tragedy and the most common cause of death worldwide. The cause of sudden cardiac death in most instances is ventricular arrhythmias such as ventricular fibrillation and ventricular tachycardia [211,212]. Connexin 43 is a main component of gap junctions in the ventricular myocardium and has a PY motif in its C-terminus. Reduced expression or altered subcellular localization of connexin 43 has been reported to be associated with contractile dysfunction, aging, and arrhythmia [213,214]. Basheer et al. reported that WWP1 targets connexin 43 for ubiquitin–proteasome degradation. Both global- and cardiomyocyte-specific overexpression of WWP1 mice die around 8 weeks of age due to lethal ventricular arrhythmia accompanied by a dramatic reduction in connexin 43. Therefore, these findings suggest an association between WWP1 and ventricular arrhythmia [187].

## 9. Drug Discovery

The most successful clinically applied drug in this field is a proteasome inhibitor for multiple myeloma [215]. However, proteasome inhibitors attenuate cardiac remodeling after pressure overload [73,216], it showed unexpected cardiac complications, including HF [217]. Therefore, the clinical application of proteasome inhibitors for cardiac diseases appears to be limited. Since E3 ligases are often focal points of cellular regulation [218,219,220], targeting E3 ligases is thought to yield higher specificity and less toxicity than other ubiquitylation cascades [221,222]. HECT-type E3 ligases have recently been described as druggable by peptide and small molecule inhibitors, and several compounds targeting NEDD4-1, NEDD subfamily, ITCH, SMURF1, E6AP, and HUWE1 have been identified [223].

Rossi et al. identified clomipramine, a commonly used drug in treating depression, as an inhibitor of ITCH, by using high-throughput screening of ITCH auto-ubiquitylation [224]. Clomipramine binds to the SH residue irreversibly in the C-lobe of the HECT domain required for ubiquitin translocation. The therapeutic potential of clomipramine in cancer has also been examined [225].

The E3 ligase activity of ITCH is modulated by several factors such as intra- and intermolecular interactions, post-translational modification, adaptor proteins, and ITCH expression itself [80,182,226,227]. Lithium is widely used as a drug for acute mania in bipolar disorder [228]. Recently, Wang X et al. demonstrated that lithium induces upregulation of ITCH and promotes ubiquitylation and degradation of Gli1, a regulator of the Hedgehog signaling pathway, in PANC-1 cells [229]. A recent report demonstrated that low-dose lithium feeding increases sarcoplasmic/endoplasmic reticulum Ca^2+^ ATPase 2a (SERCA2a) to phospholamban ratio and improved SERCA function in the murine left ventricle [230], suggesting a protective role in the heart. Although the precise mechanism by which lithium upregulates ITCH is unclear, there is a possibility that ITCH target therapy is beneficial in the heart. Further studies are required to examine whether HECT-type E3 ligase target drugs can be clinically applied to treat cardiac disease.

## 10. Limitation

This study shows the current understanding of the role of HECT-type E3 ligases in the development of cardiac disease. However, this study had several limitations. First, only a limited number of HECT-type E3 ligases have been examined to date. There have been no reports regarding the HERC subfamily in the field of cardiac disease. Second, previous studies regarding HECT-type E3 ligases in cardiac hypertrophy were limited to left ventricular hypertrophy. Recent research highlighted the importance of right ventricular hypertrophy and suggested that the ubiquitin–proteasome system is activated in the right ventricle [231]; therefore, further studies are required to elucidate the role of HCET-type E3 ligase in cardiac hypertrophy. Third, there is still no evidence for the clinical application of drugs targeting HECT-type E3 ligases in cardiac disease. However, preclinical evidence published to date supports the idea that HECT-type E3 ligases in the myocardium may be an effective interventional strategy for cardiac disease. Finally, although our understanding of HECT-type E3 ligase in cardiac disease is developing, many more are yet to be found.

## 11. Conclusions

Regulation of HECT-type E3 ligases plays an important role in determining cell fate, and HECT-type E3 ligase function is implicated in cardiac disease. Recent experimental studies have emphasized the role of HECT-type E3 ligases in the development of cardiac remodeling.

Cardiac hypertrophy and fibrosis have been proposed as important therapeutic targets in patients with HF [232], the identification of druggable targets that regulate maladaptive remodeling may provide a new avenue to control the progression of HF. HECT-type E3 ligases are often the focal point of cellular regulation; this feature makes them attractive as a therapeutic target. The continuation of research to elucidate HECT-type E3 ligases is critical for increasing our knowledge and discovering new therapeutic targets for the myriad of cardiac diseases that plague humans.

## Figures and Tables

**Table 1:** HECT-type E3 ligase and cardiac remodeling.

HECT-Type E3 Ligase	Substrate/Target	Main Findings	Reference
***Cardiac hypertrophy***			
ITCH	Dishevelled	Cardiac-specific ITCH transgenic mice inhibited maladaptive hypertrophy via Wnt/β catenin signal inhibition.	[80]
NEDD4-2	ENaC in kidney	Cardiac hypertrophy was observed in NEDD4-2 null mice on chronic high-salt diet.	[81,82]
Circular RNA WWP1	ANF and miR-23a	Circular RNA WWP1 was dysregulated in the heart treated with isoproterenol.	[83]
WWP2	PARP1	WWP2 conditional knockout mice (MycCre+;WWP2^Fl/Fl^) exacerbated isoproterenol-induced cardiac hypertrophy.	[84]
E6AP		Increased myocardium E6AP expression after pressure overload.	[16]
HUWE1	c-myc	HUWE1 conditional knockout mice spontaneously developed cardiac hypertrophy.	[85]
HACE1	Unknown	HACE1 conditional knockout mice spontaneously developed cardiac hypertrophy.	[86]
HECTD3	SUMO2/STAT1	AAV9-medited overexpression of HECTD3 inhibited pathological hypertrophy in mice.	[87]
***Cardiac fibrosis***			
WWP2	SMAD2	WWP2^mut/mut^ mice attenuated cardiac fibrosis after angiotensin II infusion and myocardial infarction.	[88]
SMURF1		SMURF1 was involved in BMP-2 antagonization for TGF-β1 signal. SMURF1 was a target of miR-10b-5p, which inhibits cardiac fibroblast activation.	[89,90]
SMURF2	SMAD7	Mediator of TGF-β signal. SMURF2 mediated SMAD7 degradation was inhibited by SMAD3 inhibitor.	[91,92]
*HFpEF*			
WWP1	Not described	Cardiac-specific overexpression of WWP1 developed cardiac hypertrophy with diastolic dysfunction.	[93]

ANF, atrial natriuretic factor; BMP, bone morphogenic protein; ENaC, epithelial Na^+^ channel; HFpEF, heart failure with preserved ejection fraction; NEDD4-2, neural precursor cell expressed developmentally downregulated 4-2; PARP1, poly(ADP-ribose)polymerase-1; SMAD, small mother against decapentaplegic; SMURF, SMAD ubiquitin regulatory factor; STAT, signal transduction and activator of transcription; SUMO2, small ubiquitin-like modifier 2.

**Table 2:** HECT-type E3 ligase and ischemia reperfusion injury, doxorubicin cardiotoxicity, and arrhythmia.

HECT-Type E3 Ligase	Substrate/Target	Main Findings	Reference
***I/R injury***			
NEDD4-1	p-Akt, PTEN	Overexpression of NEDD4-1 ameliorated myocardial apoptosis after I/R injury in rat injected with NEDD4-1 lentivirus vector.	[180]
SMURF2	EZH2	miR-322/503 ameliorated I/R injury via inhibition of SMURF2 translation.	[181]
***Doxorubicin cardiotoxicity***			
ITCH	TXNIP Unknown	Cardiac specific ITCH transgenic mice attenuated doxorubicin cardiotoxicity and myocardial infarction. miR-34b/c inhibited myocardial injury through ITCH.	[182,183]
Circular RNA ITCH	miR-17-5p miR-330-5p	Inhibition of apoptosis caused by H_2_O_2_. Inhibition of doxorubicin cardiotoxicity.	[184] [185]
***Arrhythmia***			
NEDD4-2	Nav1.5	Contribution to Nav1.5 downregulation in HF.	[186]
WWP1	Connexin 43	Cardiac-specific overexpression of WWP1 die due to ventricular arrhythmia.	[187]

H_2_O_2_, hydrogen peroxide; I/R, ischemia reperfusion injury; Nav1.5, voltage-dependent Na^+^ channel; NEDD, neural precursor cell expressed developmentally downregulated; SMAD, small mother against decapentaplegic; SMURF, SMAD ubiquitin regulatory factor; TXNIP, thioredoxin-interacting protein.

## Abbreviation

BMP	bone morphogenetic protein
CHIP	carboxyl terminus of Hsp70 interacting protein
Dvl	dishevelled
ECM	extracellular matrix
E6AP	E6-associated protein
EZH2	enhancer of zeste homolog 2
HACE	HECT domain and ankyrin repeat containing E3 ubiquitin protein ligase
HECT	homologous to E6AP C-terminus
HECTD	HECT domain E3 ubiquitin protein ligase
HERC	HECT and RLD domain containing E3 ubiquitin protein ligase
HF	heart failure
HFpEF	heart failure with preserved ejection fraction
HUWE	HECT, UBA and WWE domain containing E3 ubiquitin protein ligase
I/R	ischemia/reperfusion
JAK	Janus kinase
MDM2	mouse double mutant 2 homolog
MuRF	muscle ring finger
Nav1.5	voltage-dependent Na^+^ channel
NEDD4	neural precursor cell expressed developmentally downregulated protein 4
NFAT	nuclear factor of activated T-cells
NF-	nuclear factor kappa B
PARP1	poly(ADP-ribose) polymerase-1
PI3K	phosphatidylinositol 3-kinase
PTEN	phosphatase and tensin homolog deleted from chromosome 10
RBRs	RING-between-RINGs
RING	really interesting new genes
RLDs	regulator of chromosome condensation-like domains
SERCA2	sarcoplasmic/endoplasmic reticulum calcium ATPase 2
SMAD	small mother against decapentaplegic
SMURF	SMAD specific E3 ubiquitin regulatory factor
STAT	signal transduction and activator of transcription
SUMO2	small ubiquitin-like modifier 2
TAC	transverse aortic constriction
TGF-β	transforming growth factor beta
TRIP12	thyroid hormone receptor interactor 12
UBA	ubiquitin-associated
UBE	ubiquitin–protein ligase E3
UBR5	ubiquitin protein ligase E3 component N-recognin 5
WT	wild type
WWP	WW domain containing E3 ubiquitin protein ligase
